# Role of SMC1 in Overcoming Drug Resistance in Triple Negative Breast Cancer

**DOI:** 10.1371/journal.pone.0064338

**Published:** 2013-05-22

**Authors:** Sushma Yadav, Archana Sehrawat, Zeynep Eroglu, George Somlo, Robert Hickey, Sailee Yadav, Xueli Liu, Yogesh C. Awasthi, Sanjay Awasthi

**Affiliations:** 1 Department of Diabetes, Endocrinology and Metabolic Diseases, City of Hope Comprehensive Cancer Center, Duarte, California, United States of America; 2 Department of Molecular Biology and Immunology, University of North Texas Health Science Center, Fort Worth, Texas, United States of America; 3 Department of Medical Oncology and Therapeutics, City of Hope Comprehensive Cancer Center, Duarte, California, United States of America; 4 Division of Radiation Biology, Beckman Research Institute of City of Hope, Duarte, California, United States of America; 5 Department of Information Sciences, City of Hope, Duarte, California, United States of America; Virginia Commonwealth University, United States of America

## Abstract

Triple-negative breast cancer (TNBC) is one of the hardest subtypes of breast cancer to treat due to the heterogeneity of the disease and absence of well-defined molecular targets. Emerging evidence has shown the role of cohesin in the formation and progression of various cancers including colon and lung cancer but the role of cohesin in breast cancer remains elusive. Our data showed that structural maintenance of chromosome 1 (SMC1), a subunit of the cohesin protein complex, is differentially overexpressed both at RNA and protein level in a panel of TNBC cell lines as compared to normal epithelial or luminal breast cancer cells, suggesting that the amplified product of this normal gene may play role in tumorigenesis in TNBC. In addition, our results show that induced overexpression of SMC1 through transient transfection enhanced cell migration and anchorage independent growth while its suppression with targeted small interfering RNA (siRNA) reduced the migration ability of TNBC cells. Increased expression of SMC1 also lead to increase in the mesenchymal marker vimentin and decrease in the normal epithelial marker, E-cadherin. Immunocytochemical studies along with flow cytometry and cell fractionation showed the localization of SMC1 in the nucleus, cytoplasm and also in the plasma membrane. The knockdown of SMC1 by siRNA sensitized the TNBC cells towards a PARP inhibitor (ABT-888) and IC_50_ was approximately three fold less than ABT-888 alone. The cytotoxic effect of combination of SMC1 suppression and ABT-888 was also confirmed by the colony propagation assay. Taken together, these studies report for the first time that SMC1 is overexpressed in TNBC cells where it plays a role in cell migration and drug sensitivity, and thus provides a potential therapeutic target for this highly invasive breast cancer subtype.

## Introduction

Triple-negative breast cancer (TNBC), which comprises 15% to 20% of all breast cancers, is an aggressive breast cancer subtype with a high rate of proliferation, metastasis and poor prognosis for advanced stage disease [1]–[3]. It represents an important clinical challenge because of low or negative expression of estrogen (ER), progesterone (PR) or epidermal growth factor 2 (HER2) receptors, which comprise the currently available targets for hormonal and herceptin based therapy [4]–[6]. At present the only approved treatment for TNBC is cytotoxic chemotherapy, and TNBC patients have a much shorter disease-free survival and overall survival compared to other subtypes of breast cancer [4]–[6], [7]. Targeted therapy options has also been studied, as epidermal growth factor receptor (EGFR) expression is frequent in basal-like subtype, which comprises most TNBCs [8]. However, a randomized Phase II study of cetuximab (an anti-EGFR antibody) plus carboplatin in metastatic TNBC produced responses in fewer than 20% of patients [8]. An analysis of the triple negative subset of randomized phase III trials demonstrated progression-free survival impact of bevacizumab (an anti-VEGF antibody) but there was no evidence of overall survival impact [9]. About 20% of TNBCs are *BRCA1* (breast cancer 1, early onset) mutated, and some TNBCs demonstrate functionally *BRCA1*-mutation-like molecular characteristics and behavior; the BRCA1 protein and another enzyme, PARP (poly (ADP)-ribose polymerase) are stimulated by single- and double-strand DNA breaks and play a significant role in maintenance of genomic stability [6], [10]. As TNBCs show defects in BRCA-associated pathways, platinum compounds have shown encouraging activity due to their DNA-damage ability; the combination of platinum and PARP (poly (ADP)-ribose polymerase) inhibitors may also have improved activity in BRCA-associated breast cancer patients [11], [12]. Therefore, inhibition of PARP may render tumors lacking *BRCA* function exquisitely sensitive. The PARP inhibitor, AZD2281 (Olaparib) given to patients with *BRCA1*- and/or *BRCA2*-deficient advanced breast cancer (of which >50% were triple-negative) in a single arm study resulted in an overall response rate of 41% and progression free survival of about six months [13]. A different PARP-inhibitor ABT-888 (Veliparib) in combination with temozolomide was tested among women with advanced triple-negative breast cancer (eight had a *BRCA* mutation) in a single arm phase II study; an overall response of 7% across the entire study population increased to 37.5% in patients with *BRCA* mutations [14]. Therefore, understanding the molecular mechanisms for development of novel therapeutic agents, possibly for use in combination with agents currently shown to have some activity in TNBC, represents a high priority.

SMC1 (structural maintenance of chromosomes 1), a member of the structural maintenance of chromosomes family of ATPases binds with BRCA1 and is likely to be a component of the signaling network in which *BRCA1* maintains genomic stability [15]. It is an evolutionary conserved multifunctional protein known for its role in sister chromatid cohesion, DNA recombination and repair, and activation of the cell cycle checkpoints by ionizing radiation, ultraviolet light, and other genotoxic agents [15]–[21]. Together with SMC3, SMC1 forms a heterodimer and associates with SCC1/RAD21 and SCC3/SA to form the cohesin complex, which holds the sister chromatids from DNA replication in S-phase until chromosome separation which occurs in anaphase [15]–[21]. Emerging evidence has also shown that cohesin is involved in various other functions including transcription, cell proliferation and maintenance of pluripotency [22], [23]. SMC1 along with SMC3 has been shown to participate in microtubule-mediated intracellular transport. Cohesin-associated genes have been shown as potential drivers of tumor genomic instability; progression and mutations in various subunits of cohesin have been found in sarcoma, melanoma, colon and glioblastoma tumors [24]–[28].

In a gene expression profile of various breast cancer cell types, overexpression of both SMC1 and RAD21 was seen in MDA-MB-453, while not in MCF7 (an ER/PR positive breast cancer cell line) [29]. Additionally in breast cancer, gains at the cohesin gene chromosomal loci seem to occur more frequently at the RAD21 loci and down-regulation of RAD21 in human breast cancer cell lines was shown to increase its sensitivity to cancer chemotherapeutic agents [30], [31]. SMC3 protein present primarily in the nucleus was found in certain cell types as a secreted proteoglycan and is a component of the basement membrane of some tissues; tumor matrix and overexpression of SMC3 in NIH3T3 fibroblasts causes cell-cell contact inhibition, and display anchorage-independent growth and form foci of transformation [32], [33]. These studies have demonstrated that dysregulated expression of cohesin may play a role in cancer development and progression.

The goal of this study was to determine the expression of SMC1 in triple negative breast cancer cells, including those that are *BRCA1* mutated, as compared to normal or hormone receptor positive breast cancer cells. The role of SMC1 overexpression and suppression was tested in cell migration and anchorage-independent cell growth assay in TNBC cells. Immunocytochemical studies and flow cytometry were performed to assess the localization of SMC1 in TNBC cells. In addition, the effect of PARP inhibitor, ABT-888 (Veliparib) was tested alone and in combination with SMC1 siRNA in normal and TNBC cells including *BRCA1* mutated cell lines.

## Materials and Methods

### Reagents

cDNA of SMC1 (3701 bp) was kindly provided by Dr. John Schmiesing, Department of Biological Chemistry, University of California, Irvine, CA. Bacterial strains [DH5α and BL21(DE3)], high fidelity Taq DNA polymerase and dNTPs were purchased from Invitrogen Life Tech. (Carlsbad, CA). pET30a(+), the T7 promoter based expression vector was purchased from Novagen Inc. (Madison, WI). DNA ligase and restriction enzymes was from New England Biolabs (Beverly, MA). The primers and duplex siRNA were synthesized by Biosynthesis Inc. (Lewisville, TX) and Integrated DNA Technology (Coralville, IA). A 60-mer shRNA for SMC1 was designed using Oligoengine 2.0 and non-targeted scrambled shRNA and SMC1 shRNA were purchased from Oligoengine (Seattle, WA). The DeadEnd™ fluorometric TUNEL system was from Promega (Madison, WI). ABT-888 (Veliparib) was obtained from Chemie-Tek (Indianapolis, IN). Polyclonal rabbit-anti-human SMC1 IgG was purchased from Bethyl Laboratories (Montgomery, TX). Sepharose A, horseradish peroxidase (HRP)-conjugated anti-mouse, anti-rabbit and anti-goat secondary antibodies were procured from Sigma (St. Louis, MO). Rhodamine red-x-conjugated and FITC-conjugated goat-anti-rabbit IgG were from Thermo Scientific (Rockford, IL). Vimentin, E-cadherin, and GAPDH antibodies and ECL reagent were purchased from Cell Signaling Technology (Danvers, MA). Anti-SMC3 IgG was from Santa Cruz Biotechnology (Santa Cruz, CA). Subcellular protein fractionation kit was from Thermo Scientific (Rockford, IL). Lipofectamine 2000 and Lipofectamine RNAiMax transfection reagent kit were purchased from Invitrogen Inc. (Grand Island, NY). Sources of other reagents were the same as previously described [34].

### Cell lines and cultures

A human non-tumorigenic mammary epithelial (MCF10a), ER+/hormone responsive luminal epithelial (MCF7) and a series of TNBC cell lines (hs578T, MDA-MB-231, MDA-MB-468, HCC1937) were from American Type Culture Collection (ATCC) and provided by Drs. Shiua Chen and Susan Kane, City of Hope (Duarte, CA). MDA-MB-436 (from ATCC) was provided by Dr. Linda Malkas, City of Hope (Duarte, CA); human umbilical vascular epithelial (HUVEC) cells were purchased from ATCC (Manassas, VA). All cells were cultured at 37°C in a humidified atmosphere of 5% CO_2_ in the appropriate medium: DMEM/F12 with 15 mM Hepes buffer, 5% horse serum, 10 µg/ml insulin, 20 ng/ml EGF, 100 ng/ml cholera toxin, 0.5 µg/ml hydrocortisone (MCF10a), MEM with MEM vitamin, 15 mM Hepes buffer, Sodium bicarbonate (MDA-MB-436), RPMI-1640 (MCF7) and DMEM (hs578T, MDA-MB-231, MDA-MB-468, HCC1937, HUVEC), medium supplemented with 10% fetal bovine serum (FBS) and 1% penicillin/streptomycin (P/S) solution.

### Cloning and generation of SMC1 expression vectors

The cDNA of SMC1 was used as a template for PCR amplification of the SMC1 coding sequence. The upstream (5′ GGCGGATCCATGGGGTTCCTGAAACTGAT 3′) and downstream (5′ CCGCTCGAGCTACTGCTCATTGGGGTT 3′) primers were designed to introduce a BamH1 restriction site (underlined) immediately upstream of the initiator codon and XhoI site (underlined) immediately downstream of the stop codon of SMC1 open reading frame. The PCR amplification was performed under following incubation conditions: DNA template 500 ng, primers 30 pmol each, dNTPs 0.2 mM each, high fidelity PCR buffer 1X, MgSO_4_ 2 mM and platinum Taq high fidelity 2.05 units. PCR cycles were as follows: 94°C for 5 min followed by 35 cycles of 94°C for 30 s, followed by 60°C for 30 s and 2 min at 68°C and a final extension at 68°C for 7 min. PCR product was purified by using Qiagen PCR purification kit and digested with BamHI/XhoI restriction enzymes. The cleaved PCR products were ligated into pET30a(+) and pcDNA3.1 previously digested with the same restriction enzymes and the ligated products were expressed into the DH5α competent cells and plasmid DNA was purified from the overnight culture of single colony using Qiagen DNA purification kit. Techniques for restriction enzyme digestion, ligation, transformation and other standard molecular biology manipulations were based on methods described by Sambrook et al. [35]. The sequence of the SMC1 was confirmed by DNA sequencing. Following verification of the sequence, the pcDNA3.1/SMC1 was used for transfection and pET30a(+)/SMC1 was used to transform *E. coli* strain BL21(DE3) and protein was expressed in *E. coli* BL21(DE3) grown at 37°C after induction with 0.4 mM IPTG.

### Purification of recombinant SMC1 by DNPSG -affinity chromatography and reconstitution into proteoliposomes

We purified SMC1 to near homogeneity using dinitrophenyl-S-glutathione (DNP-SG) affinity resin prepared as previously described by us [36]–[38]. Briefly, bacteria was lysed in the presence of polidocanol (C_12_E_9_) 1% (w/v) in lysis buffer containing 10 mM Tris-HC1 pH 7.4, 1.4 mM ß-mercaptoethanol, 100 µM EDTA, 50 µM BHT, and 100 µM PMSF, sonicated three times in ice for 30 s each and incubated in the above buffer for ∼4 h with gentle shaking, followed by centrifugation at 27, 000× g for 30 min. SMC1 was purified by binding the resulting membranes to DNP-SG Sepharose 4B affinity resin followed by removal of contaminating proteins and elution with 10 mM ATP, 10 mM MgCl_2_ and 0.025% polidocanol (C_12_E_9_) in lysis buffer as described previously [36]–[38]. The eluate was concentrated using the Amicon Centriprep concentrator, followed by sequential dialysis against lysis buffer containing 2% (v/v) DE-52 and lysis buffer containing 1% (w/v) Chelex® resin for 24 h each with two buffer exchanges of 2 L each. Since polidocanol interferes with Bradford reagent, protein was estimated by the method of Minamide and Bamburg [39]. Protein was quantified by SDS-PAGE and western blots using rabbit-anti-human SMC1 IgG. Purified SMC1 was reconstituted into functional liposomes as described by us [36]–[38].

### Subcellular fractionation and immunoprecipitation

Subcellular distribution of SMC1 was determined in the MDA-MB-231 breast cancer cells using subcellular protein fractionation kit (Thermo Scientific) following manufactures directions. Briefly, about 2×10^6^ MDA-MB-231 cells were harvested with trypsin-EDTA and then centrifuged at 500× g for 5 min and washed with ice-cold PBS. The cell pellet was re-suspended in 0.2 mL CEB and incubated for 10 min at 4°C with gentle shaking and was centrifuged at 500× g for 5 min to separate cytoplasmic fraction from the pellet (cytoplasmic extract). The pellet was resuspended in MEB containing protease inhibitors, vortexed and incubated at 4°C for 10 min and centrifuged at 3,000× g for 10 min to isolate plasma membrane in the pellet (fraction PM). The pellet was dissolved in ice-cold NEB-containing protease inhibitor, CaCl_2_ and monococcal nuclease and by centrifugation at 16, 000× g for 5 min. The total protein in each fraction was quantified by modified Bradford's method and equal concentrations of protein (50 µg) were loaded on SDS-PAGE.

For immunoprecipitation, 100 µl of protein-A Sepharose bead slurry (50%)/mL of cell lysate was added and incubated at 4°C for 10 min on a rocker, followed by centrifugation at 14,000× g at 4°C for 10 min, and the concentration of protein in the cell lysate was determined by Bradford's assay [39]. The cell lysate (∼1 mg/mL) was incubated with anti-SMC1 IgG overnight at 4°C on a rocker and the immune-complex was captured by adding 100 µL protein-A Sepharose bead slurry with gently rocking on an orbital shaker at 4°C. The Sepharose beads were collected by pulse centrifugation for 5 sec in a micro-centrifuge at 14,000× g. The supernatant fraction was discarded and beads were washed 3 times with ice-cold RIPA buffer. Sepharose beads were resuspended in sample buffer and boiled for 5 minute. The beads were collected by centrifugation and the supernatant was loaded on the SDS-PAGE and were characterized by western blot using anti-SMC1 and anti-SMC3 IgG.

### Membrane localization of SMC1 by FACS analysis

Cell-surface localization of SMC1 was performed using indirect flow cytometry protocol as described [40]. Briefly, MDA-MB-231 cells were harvested and suspended in approximately 1×10^6^ cells/mL in ice cold PBS, containing 10% FBS and 1% sodium azide. Cells were incubated with anti-SMC1 IgG (1 µg/mL) in 3% BSA/PBS solution at 4°C for 2 h, followed by washing with PBS and incubated with FITC-conjugated secondary antibody for 30 min at room temperature in dark. Cells were washed with PBS, resuspended in ice-cold PBS containing 3% BSA and 1% sodium azide, and analyzed by CyAn™ advanced digital processing flow cytometer at analytical cytometry core facility, City of Hope.

### Immuncytochemical localization of SMC1 in breast cancer cells

Immunocytochemical localization of SMC1 was performed on MDA-MB-231 cells by method described previously with slight modifications [38], [41]. Cells (∼0.4×10^6^ cells) were grown on sterilized glass cover-slips in 12 well plates. After 24 h, cells were fixed with ice-cold methanol and acetic acid (3∶1). Nonspecific antibody interactions were minimized by pre-treating the cells with 10% goat serum in PBS for 60 min at room temperature. Subsequently, rabbit-anti-human-SMC1 IgG (1∶500 dilution) was added and incubated overnight at 4°C in a humidified chamber. After washing with PBS four to five times, the cells were subjected to Rhodamine red-x-conjugated goat-anti-rabbit IgG for 2 h at room temperature in a humidified chamber, followed by washing with PBS five times. DAPI (4′,6-Diamidino-2-phenylindole) was used as a nuclear counter-stain. Finally, cover slips were removed and dried in air and mounted on slides upside down with Vectashield mounting medium. Slides were analyzed by laser scanning confocal microscope (Zeiss LSM510 META, Germany).

### Transfection of SMC1 in breast cancer cells

SMC1 cDNA was sub-cloned into eukaryotic expression vector pcDNA3.1 (Invitrogen). MDA-MB-231 cells were transiently transfected with the eukaryotic expression vector alone (pcDNA3.1) or with pcDNA3.1/SMCl using Lipofectamine 2000 according to manufacturer's instructions. Expression of SMC1 mRNA was evaluated by RT-PCR analysis. RNA prepared using Trizol-reagent (Invitrogen) was quantified and purity determined by measuring absorbance at 260 and 280 nm using a nano-drop spectrophotometer (Thermo Scientific). SMC1 gene specific primers [307–326 bp upstream primer, 5′: GTCAGCATGGTCTACTCTGA and 730–750 bp downstream primer, 5′: CTTAAAGAGCTGCAGCTGTAC] were used for RT-PCR using Superscript^III^ one step RT-PCR kit (Invitrogen).

### SMC1 siRNA and shRNA preparation

The targeted cDNA sequence for SMC1: 5′-GCAATGCCCTTGTCTGTGA corresponds to nt 1910–1928 in the open reading frame. Selected DNA sequence was subjected to blast-search (NCBI database) against EST libraries, to ensure that only SMC1 gene was targeted. The corresponding siRNA sequence was GCAAUGCCCUUGUCUGUGAdTdT and UCACAGACAAGGGCAUUGCdTdT. A 21 nucleotide long scrambled siRNA was used as a control. The sequence of the scrambled siRNA was CAUCGAAAUCGUUGCAGUUACdTdT and GUAACUGCAACGAUUUCGAUGdTdT. Chemically synthesized duplex siRNA in desalted form was purchased from Biosynthesis Inc. and Integrated DNA Biotechnology. A 60-mer shRNA for SMC1 was designed using the oligoengine 2.0 software and asymmetric annealed oligonucleotide containing the ends compatible with digestion using Bgl II/Hind III were cloned in pSUPER-neo-gfp vector. The sequences for oligos were: GATCCCCAATGACCCATTTCACGAAGTTCAAGAGTCTTCTGTAAATGGGTCATTTTTTTA with Bgl II site underlined and AGCTTAAAAAAATGACCCATTTCACGAAGACTCTTGAACTTCTGTAAATGGGTCATTGGG with Hind III site underlined. The oligos were annealed as per manufactures directions, digested with Bgl II and Hind III restriction enzymes and clones into pSUPER-neo-gfp vector digested with the same enzymes. Transfection of siRNA and shRNA was performed using the Lipofectamine RNAiMax transfection reagent (Invitrogen) following manufactures instructions, and assayed for silencing at 24 and 48 hours after transfection.

### Effect of SMC1 overexpression and suppression on colony propagation

MDA-MB-231 cells (0.1×10^6^ cells/500 µL) untreated and treated with control-liposome, SMC1-liposomes (each liposomes final purified SMC1 protein conc. 40 µg/mL), as well as transfected with scrambled and SMC1 siRNA (50 nM), control vector (pcDNA3.1) and pcDNA3.1/SMC1 as described above. After 24 hour, aliquots of 50 and 100 µL were taken in 60 mm Petri dishes, separately; in a total volume of 4 mL with medium in each Petri dish and were incubated in CO_2_ incubator for 10 days with changing the medium every two days. After 10 days cells were stained with 0.5% methylene blue for 30 min and colonies were counted using Innotech Alpha Imager [38], [41], [42].

### Anchorage-independent growth

Anchorage independent growth assay was performed in MDA-MB-231 cells transfected with scrambled shRNA or SMC1 shRNA in soft agar cell culture in a 10-cm plate. Briefly, a 0.6% agar/DMEM pre-layer (8 mL) was poured in a 10-cm dish. After solidification, semisolid feeder layer (a mixture of pre-warmed (37°C) full growth DMEM and 0.6% soft agar were mixed with 3×10^3^ cells) was overlayed on the solidified layers. Cells were allowed to grow in the humidified 37°C incubator with 5% CO_2_ for 21 days and colony numbers were determined by Innotech alpha imager HP.

### Wound healing Assay

MDA-MB- 231 cells were seeded in 12 well plates until they reached 60–70% confluency, followed by transfection with control vector (pcDNA3.1), pcDNA3.1/SMC1, scrambled siRNA or siRNA against SMC1. After 24 hours, cell monolayer was scraped in a straight line to create a “scratch” with a p200 pipet tip [43], [44]. The cells were washed with growth medium to remove debris and to smooth the edge of the scratch, then replaced with 1 mL growth medium and incubated for 24 h at 37°C in 5% CO_2_. Images were taken at 0 and 24 hour by placing the 12 well plate under phase contrast microscope (Olympus IX81 automated Inverted) using automated Stage-Pro program to ensure the same area is aligned and photographed. Migrated cells in the wound area at 24 hour were counted from five different fields and expressed as the means ± S.E. of three independent experiments.

### Effect of SMC1 overexpression and suppression on markers of cell migration

MDA-MB-231 cells were seeded in 6 well plates and when they were about 75% confluence, transfected with pcDNA3.1, SMC1/pcDNA3.1, scrambled siRNA and SMC1 siRNA as described above. After 24 hour, cells were harvested and expression of vimentin and E-cadherin were determined by western blot analysis. Cell extracts containing 50 µg proteins were separated on SDS-polyacrylamide gels (12.5%) and transferred onto nitrocellulose (Bio-Rad) membranes. The membranes were blocked with 5% fat-free milk in PBS at room temperature for 30 min and incubated overnight at 4°C with the appropriate primary antibody in 5% milk in PBS. The membranes were then incubated with the appropriate secondary antibodies at room temperature for 1 h, washed with PBS, treated with ECL-chemiluminescence reagent as per the manufacturer's instructions, and exposed to ECL film at room temperature. The bands were quantified by densitometry using Innotech Alpha Imager.

### Effect of SMC1 on cell apoptosis by TUNEL assay

Aliquots of cells (0.1×10^6^ cells) were placed into 12 well plates containing glass cover-slips. After 24 hour incubation with medium, the cells were transfected with 50 nM SMC1-siRNA or scrambled-siRNA and SMC1/pcDNA3.1 vector using Lipofectamine RNAiMax and Lipofectamine 2000 transfection reagent following manufactures instructions. Apoptosis was determined by the labeling of DNA fragments with terminal deoxynucleotidyl-transferase dUTP nick-end labeling (TUNEL) assay using Promega apoptosis detection system according to the protocol provided by manufacturer. The total number of cells (blue) and apoptotic cells (green) were counted using the Image-Pro 3 software and % of apoptotic cells was calculated.

### Effect of SMC1 siRNA in sensitizing ABT-888 by cell survival and colony forming assay

The drug sensitivity assay was performed in a number of TNBC cells including basal-like (MDA-MB-468, HCC1937) and mesenchymal stem-like (MDA-MB-231, MDA-MB-436) subtypes and a human umbilical vein endothelial cells (HUVEC) by MTT (3-[4,5-dimethylthiazol-2-yl]-2,5 diphenyl tetrazolium bromide) assay as described [41], [42]. Briefly, approximately 2×10^6^ cells were plated into 100 mm culture plate and transfected with 50 nM SMC1 siRNA or scrambled siRNA in Lipofectamine RNAiMax transfection reagent following manufactures instructions and approx. 20,000 transfected cells were plated into each well of 96 well flat-bottomed microtiter plates. After 24 hour incubation at 37°C, the cells were treated with increasing concentrations of ABT-888 and cell survival was measured by performing MTT assay 72 hour later as described previously [41], [42]. Briefly, after 72 hour, 20 µl of 5 mg/ml MTT was introduced to each well and incubated for 2 hour, the plates were then centrifuged and medium was decanted. Cells were subsequently dissolved in 100 µl DMSO with gentle shaking for 2 hour at room temperature, followed by measurement of OD_570_ nm [42]. Four replicate wells were used in each point in each of three separate measurements. Measured absorbance values were directly linked with a spreadsheet for calculation of IC_50_, defined as the drug concentration that reduced formazan formation by 50%.

To further validate the effect of SMC1 silencing on the efficacy of ABT-888 in TNBC, 0.1×10^6^ cells/500 µL cells (MDA-MB-468, HCC1937, MDA-MB-231 and MDA-MB-436) were transfected with scrambled siRNA or SMC1 siRNA as described above. Aliquots 50 and 100 µL was added to 60 mm tissue culture treated petri dishes, separately and 4 ml growth medium containing ABT-888 enough to kill 50% cells calculated by cell survival assay was added with changing the medium containing drug every 2 days. After 10 days, cells were stained with 0.5% methylene blue for 30 min and colonies were counted using Innotech Alpha Imager [42].

### Statistical Analyses

All data were summarized as the mean ± SD. Further, we evaluated significance of differences between control and treatment groups using a two-tailed unpaired Student's t-test. Differences were considered statistically significant when the *p* value was less than 0.05. All statistical analysis was carried out in the freely downloadable software R (http://cran.r-project.org/).

## Results

### SMC1 expression is abnormally elevated in breast cancer cell lines

**Figure 1:**
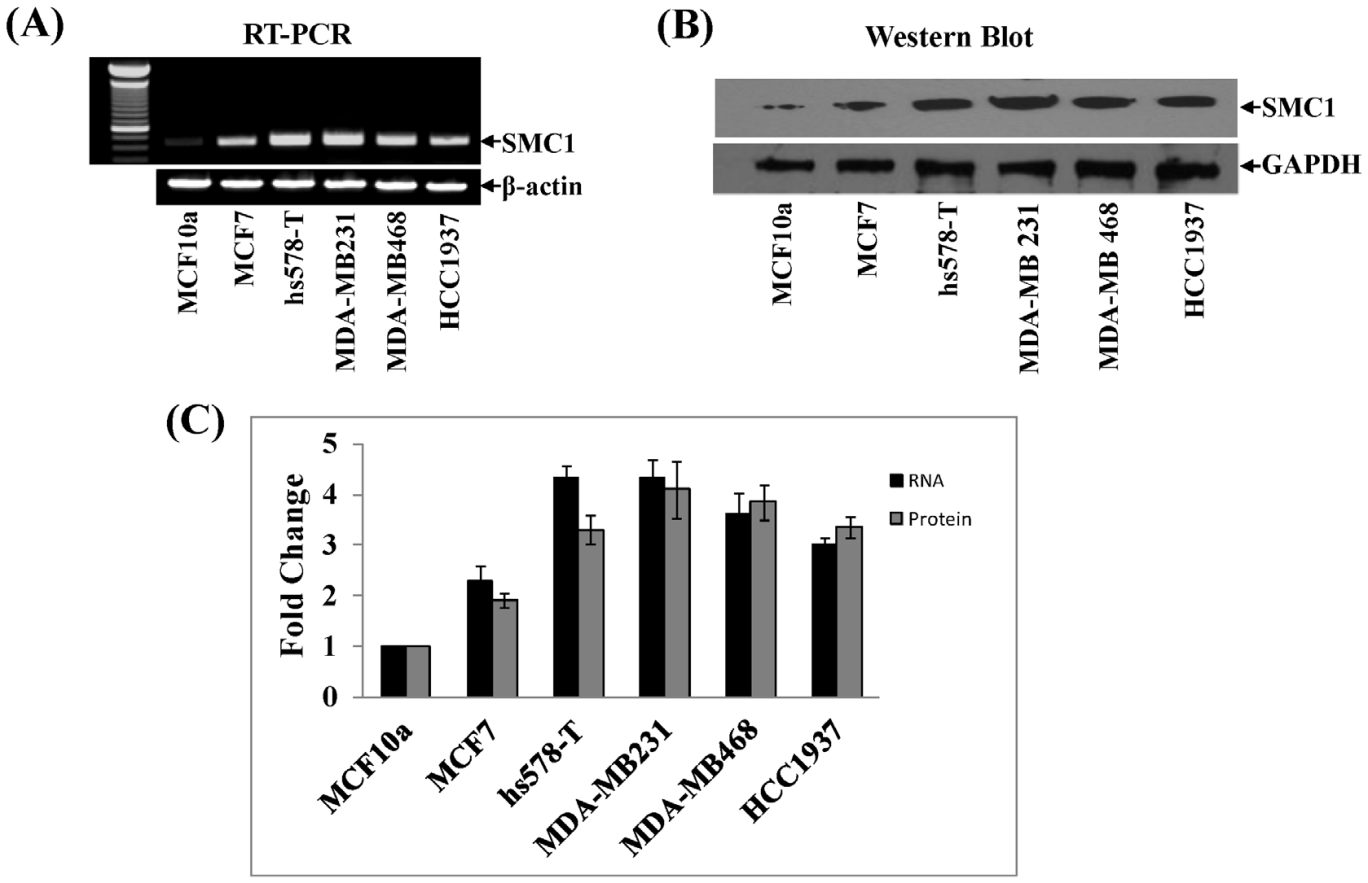
The expression of SMC1 in different breast cancer cells. Expression of SMC1 was determined at the gene (RT-PCR) and protein level (western blot). For SMC1 gene expression, total RNA was purified using Trizol reagent (Invitrogen) as detailed in the methods section and expression of SMC1 mRNA was evaluated by RT-PCR analysis (**Panel A**) using gene specific primers [307–326 bp (upstream primer) and 730–750 bp (downstream primer)]. ß-actin was used as an internal control. SMC1 protein expression was evaluated by western blot analysis. Briefly, crude cell homogenate was prepared in RIPA buffer as described in method section and crude protein (50 µg) was subjected to SDS-PAGE and western blots using rabbit-anti-human SMC1 IgG (**Panel B**). Western blot was developed by ECL- reagent (Cell Signaling). GAPDH was used as an internal control. The level of SMC1 gene and protein was quantified by densitometry and was plotted as a fold change normalized to non-tumorigenic mammary epithelial breast cell, MCF10a (**Panel C**).

The expression of SMC1 was checked at both RNA (RT-PCR) and protein (western blot) level in a panel of TNBC cell lines (MDA-MB-231, hs578, MDA-MB-468, MDA-MB-436 and HCC1937) and compared with a ER+/hormone responsive luminal epithelial breast cancer cell (MCF7) and a non-tumorigenic mammary epithelial (MCF10a) cell line (**Table 1**). The mRNA expression of SMC1 was higher in all the TNBC cell lines including *BRCA1* mutated TNBC lines as compared to MCF7 or MCF10a cells (Fig. 1A). Expression of SMC1 protein was confirmed by western blot against rabbit-anti-human-SMC1 IgG (Fig. 1B). The RT-PCR and western blot bands quantified by densitometry showed a 3–4 fold increase in the expression of SMC1 at the RNA as well as protein level in TNBC cells (Fig. 1C). These studies showed that SMC1 is differentially overexpressed in breast cancer cells, particularly in TNBC cell lines, including *BRCA1* mutated lines.

**Table 1:** Expression of SMC1 in breast cancer and non-tumorigenic breast epithelial cells.

Cell Line	TNBC*	Histology*	Mutations*	SMC1 expression RNA	SMC1 expression protein
MCF10a	No	Non-tumorigenic, epithelial	NA	1	1
MCF7	No ER/PR+	Ductal carcinoma	NA	1.8±0.28	2.3±0.14
Hs-578T	Mesenchymal stem like	Carcinosarcoma	CDKN2A, HRAS,TP53	4.3±0.21	3.3±0.28
MDA-MB-231	Mesenchymal stem like	Ductal carcinoma	BRAP, CDKN2A, KRAS, NF2, P53	4.3±0.35	4.1±0.57
MDA-MB-468	Basal-like 1	Ductal carcinoma	PTEN, RB1, SMAD4, P53	3.6±0.42	3.8±0.35
HCC1937	Basal-like 1	Ductal carcinoma	BRCA1, P53, MAPK13, MDC1	2.9±0.14	3.2±0.21
MDA-MB-436	Mesenchymal stem like	Invasive ductal carcinoma	BRCA1, TP53	3.6±0.35	3.0±0.28

* From Lehmann et al. [53].

### Intracellular localization of SMC1

**Figure 2:**
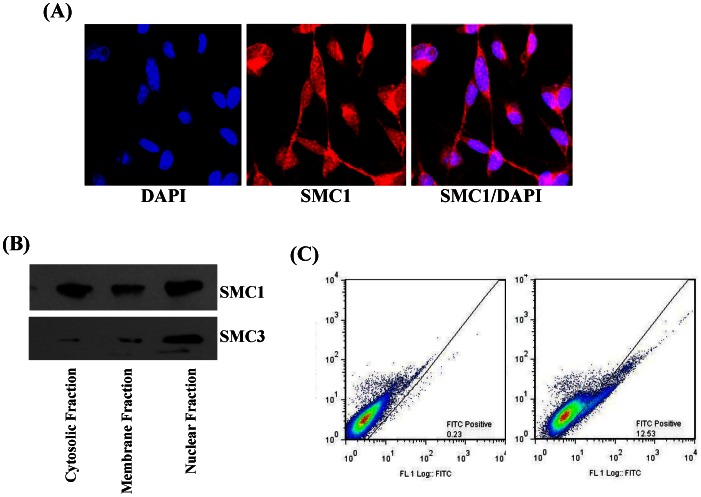
Cellular localization of SMC1 in MDA-MB-231 Cells. Immunocytological localization SMC1 was performed on MDA-MB-231 fixed cells by method described previously with slight modifications [37], [38], [40], [41]. Cells were grown on glass cover slips and fixed with ice-cold methanol and acetic acid (3∶1). Nonspecific antibody interactions were minimized by pre-treating the cells with 10% goat serum in PBS for 60 min at room temperature. The cells were subjected to immuno-cytochemistry using anti-SMC1 IgG (raised in rabbit) as a primary antibody and goat-anti-rabbit Rhodamine red-x-conjugated secondary antibody. DAPI (4′, 6-Diamidino-2-phenylindole) was used as a nuclear counter-stain. Slides were analyzed by confocal laser microscope (Zeiss LSM510 META, Germany) at 40× magnification (**Panel A**). Surface localization of SMC1 was determined by flow cytometry using indirect flow cytometry protocol [40]. Briefly, MDA-MB-231 cells were harvested and resuspended to approximately 1×10^6^ cell/ml in ice-cold PBS, 10% FBA and 1% sodium azide. Cells were incubated with 1 µg/ml anti-SMC1 IgG in 3% BSA/PBS solution and incubated at 4°C for 2 hour followed by washing with PBS and incubated with FITC-conjugated secondary antibody for 30 min at room temperature in dark. Cells were washed with PBS 3–5 times and resuspended in ice-cold PBS containing 3% BSA and 1% sodium azide and analyze by flow-cytometry (**Panel B**). Subcellular distribution of SMC1 was determined in the MDA-MB-231 cells using subcellular protein fractionation kit (Thermo Scientific) as detailed in methods section. Immuno-precipitation was performed in all the 3 fractions using anti-SMC1 IgG using the protocol as described in method section. All the immune-precipitates were characterized by western blot using anti-SMC1 and anti-SMC3 IgG (**Panel C**).

Immunocytochemical localization of SMC1 was performed in MDA-MB-231 cells using anti-SMC1 antibodies and analyzed by confocal fluorescence microscopy. SMC1 was found to be present in the nucleus, cytosol and surprisingly on the plasma membrane (Fig. 2A). Subcellular distribution analysis of SMC1 in MDA-MB-231 cells was performed using a subcellular protein fractionation kit (Thermo Scientific) which confirmed the presence of SMC1 in the nucleus, cytosol as well as the plasma membrane fraction (Fig. 2B).

Since SMC1 is known to form a complex with SMC3, we examined whether this interaction occurs in the MDA-MB-231 cells. Anti-SMC1 IgG was used for immunoprecipitation, and the immunoprecipitate was examined using anti-SMC1 and anti-SMC3 antibodies in Western blots. Results of these studies showed that anti-SMC1 IgG could precipitate both SMC1 and SMC3 in all three fractions (cytosolic, membrane and nuclear), and confirmed that the largest amount of SMC1-SMC3 complex was in the nuclear fraction. The membrane localization of SMC1 was further confirmed by flow-cytometry using anti-SMC1 as primary and FITC-conjugated anti-IgG secondary antibodies. Our results showed that SMC1 was indeed detectable on the cell surface, which accounted for ∼15% of total cellular SMC1 in these cells (Fig. 2C). Taken together, these results clearly show that SMC1 is present in the nucleus as well as in the cytoplasm and plasma membrane.

### Effect of SMC1 on colony propagation and cell transformation

**Figure 3:**
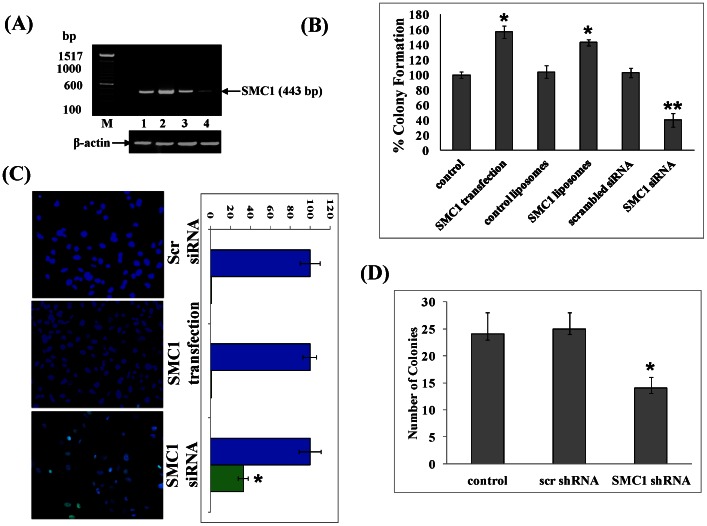
Effect of SMC1 on cellular Viability and Anchorage Independent Cell Growth. The MDA-MB-231 cells were transfected with the eukaryotic expression vector alone (pcDNA3.1) and SMC1/pcDNA3.1, using Lipofectamine 2000 transfection reagent (Invitrogen). Transfection of scrambled and SMC1 siRNA was performed with Lipofectamine RNAiMax kit (Invitrogen) following manufactures instructions. Total RNA was purified using Trizol reagent and quantified using a nano-drop spectrophotometer (Thermo Scientific). Expression of SMC1 mRNA was evaluated by RT-PCR analysis using gene specific primers [nt 307–326 bp (upstream primer) and nt 730–750 bp (downstream primer)] and β-actin was used as a control. Equal amount of DNA was loaded on 1% agarose gel; lane 1, pcDNA3.1 (control vector); lane 2, pcDNA3.1/SMC1; lane 3, scrambled siRNA; and lane 4 SMC1 siRNA to check for the overexpression and silencing of SMC1(**Panel A**). For SMC1-liposomes, SMC1 was purified by DNP-SG affinity chromatography and reconstituted into liposomes using our established procedure [36], [38]. Viability of colonies was determined by colony forming assay, performed in untreated, control-liposomes, SMC1-liposomes, scrambled and SMC1 siRNA, pcDNA3.1 and SMC1/pcDNA3.1 transfected MDA-MB-231 cells (0.1×10^6^ cells/500 µl in triplicates). Aliquots of 50 and 100 µl (in triplicates) were taken in 60 mm size petri-dishes, separately, in a total volume of 4 ml with medium. After 10 days, cells were stained with 0.5% methylene blue for 30 min and colonies were counted using Innotech Alpha Imager. The results shown are normalized to control untreated cells. Values are means ± S.D. of three separate experiments (**Panel B**). The effect of SMC1 overexpression (SMC1 transfected) and suppression (SMC1 siRNA) was also checked on the cell apoptosis by using TUNEL apoptosis kit (Promega). Briefly, approximately 0.1×10^6^ MDA-MB-231 cells were grown on the cover slips in 12 well plate and transfected with SMC1/pcDNA3.1 and SMC1 siRNA as described in method section. TUNEL apoptosis assay was performed using the Promega Apoptosis Detection Kit according to manufactures instructions. The slides were analyzed by fluorescence microscope (Olympus IX81 automated Inverted) using a standard fluorescein filter set to view the green fluorescence at 520 nm, and blue fluorescence at >340 nm. Photographs taken at identical exposure at 20× magnification are presented. Apoptotic cells showed green fluorescence and characteristic cell shrinkage (**Panel C**). MDA-MB-231 cells transfected with scrambled shRNA and SMC1 shRNA were also tested for the anchorage-independent growth on soft agar as described in the methods section and colonies were counted after 21 days and plotted (**Panel D**).

The effect of modulating cellular SMC1 on the colony forming activity of MDA-MB-231 cells was examined using either liposomal delivery of purified recombinant SMC1 or transient transfection of SMC1-pcDNA3.1 plasmid to augment the level of SMC1, or using SMC1 siRNA to deplete cellular SMC1. Recombinant human SMC1 protein was purified from bacterial cultures of BL21(DE3) expressing the pET30a(+)/SMC1 plasmid using DNP-SG affinity chromatography and reconstituted in artificial liposomes consisting of asolectin-cholesterol (4∶1) using established procedures [36]–[38]. Control liposomes were prepared in the absence of purified SMC1. Cells were treated with SMC1-liposomes (40 µg/ml SMC1 protein) or control-liposomes for 24 hour followed by removing medium and washing 3 times with PBS. Successful augmentation of cellular SMC1 in cells treated with SMC1-proteoliposomes was evident from western blots. The alternative approach for augmentation of SMC1 used in present studies was transient transfection of pcDNA3.1/SMC1 eukaryotic expression vector into MDA-MB-231 cells. Control cells were transfected with empty pcDNA3.1 vector. Successful transfection was confirmed by RT-PCR and depletion of SMC1 by SMC1 siRNA was also successfully achieved as demonstrated by RT-PCR (Fig. 3A). Augmenting SMC1 using SMC1 proteoliposomes or transient transfection significantly increased the clonogenic potential of cells (50% and 60%, respectively, p<0.01) (Fig. 3B), and depletion of SMC1 by siRNA caused decrease in colony formation by 60% (p<0.01). TUNEL-assay confirmed that depletion of SMC1 causes more than 50% apoptosis in MDA-MB-231 cells (Fig. 3C). The suppressive effect of SMC1 depletion was confirmed in an anchorage independent cell growth assay in which shRNA was used to deplete SMC1 (Fig. 3D). Taken together, these results indicate that cellular SMC1 level contributes to the growth and survival of MDA-MB-231 cells and its depletion reduces clonogenic potential and anchorage-independent growth, and increases susceptibility to apoptosis.

### Effect of SMC1 on cell migration

**Figure 4:**
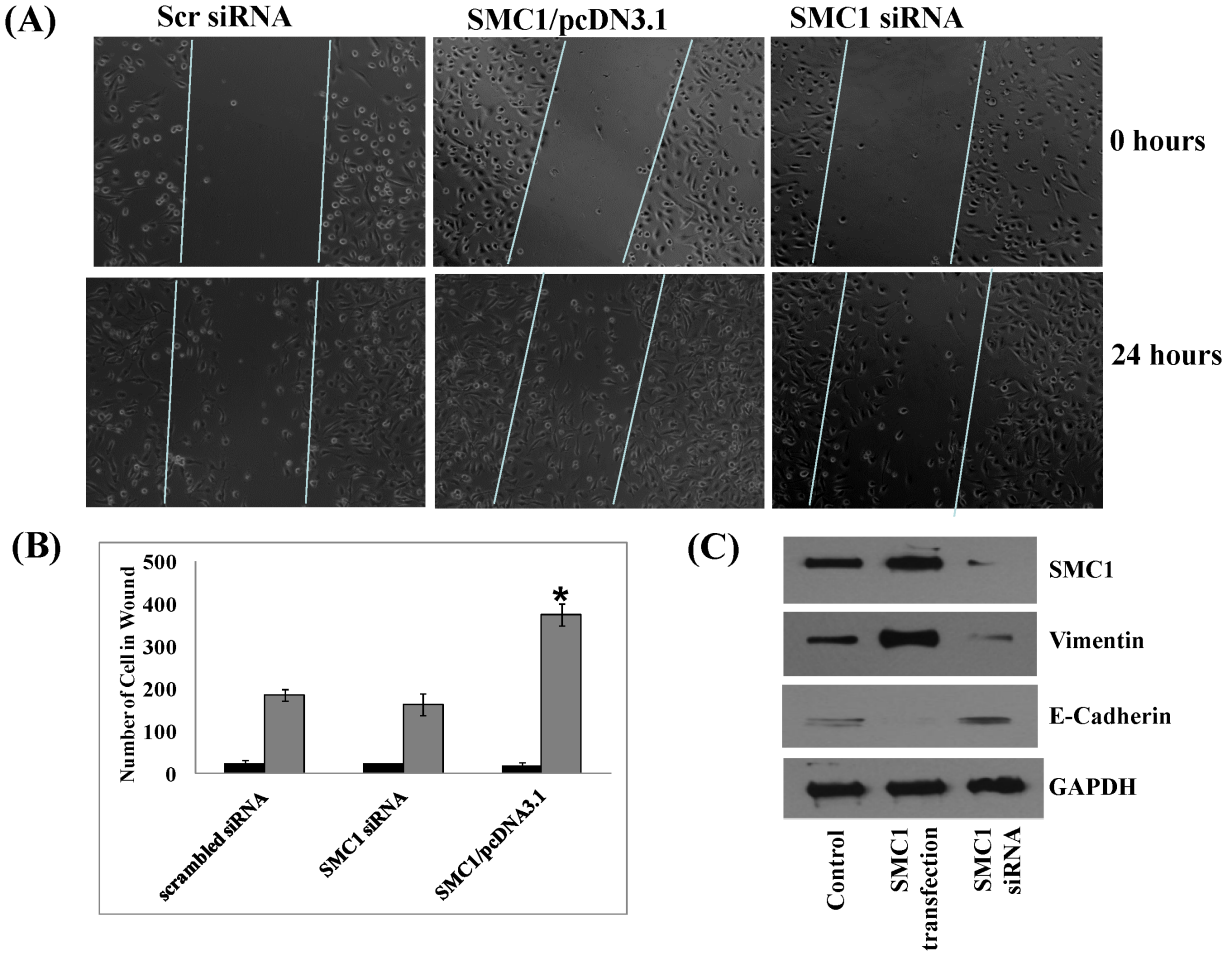
Role of SMC1 in Cell Migration. Scratch wound assays were performed in MDA-MB-231 cells transfected with scrambled siRNA (scr siRNA) or siRNA against SMC1, control vector (pcDNA3.1), and SMC1/pcDNA3.1. After 24 hours, confluent monolayers of cells were wounded, and healing of the wound by cell migration was monitored for 24 hour. Images were taken at 0 and 24 hours (**Panel A**). Migrated cells in the wound area at 24 hour were counted from five different fields and expressed as the means ± S.E. of three independent experiments (**Panel B**). The effect of SMC1 overexpression (SMC1 transfected, lane 2) and suppression (SMC1 siRNA, lane 3) was also determined by checking the expression of vimentin and E-cadherin, markers of angiogenesis and metastasis by western blot analysis (**Panel C**). As can be seen in figure 5 (**Panel C**), there was overexpression of vimentin in SMC1 transfected MDA-MB-231 cells and suppression of E-cadherin as compared to control while on SMC1 suppression, there was suppression of vimentin while no significant change in the expression of E-cadherin.

The effect of SMC1 overexpression or suppression on cell-migration was examined using an established scratch assay [43], [44]. Cells transfected with SMC1 were able to migrate efficiently and cover nearly all of the wounded area within 24 hours, whereas cells transfected with SMC1 siRNA were much less efficient in this process as compared to the control cells (Fig. 4A). The colonies counted in the scratch area after 24 hours were plotted and there was twofold increase in the wound healing capacity in SMC1 transfected cells (Fig. 4B). SMC1 overexpression also increased the level of vimentin, a major intermediate filament protein which regulates epithelial–mesenchymal transition, (an essential process during oncogenic transformation as well as metastasis) and reduced the level of E-cadherin (a protein required to maintain the epithelial phenotype of the basement membrane) (Fig. 4C). The expression of vimentin is associated with enhanced motility of tumor cells and hence overexpression of vimentin by enhanced expression of SMC1 shows its role in maintenance of cell adhesion and metastasis.

### Effect of combining a PARP-inhibitor with SMC1 siRNA in TNBC cells

**Figure 5:**
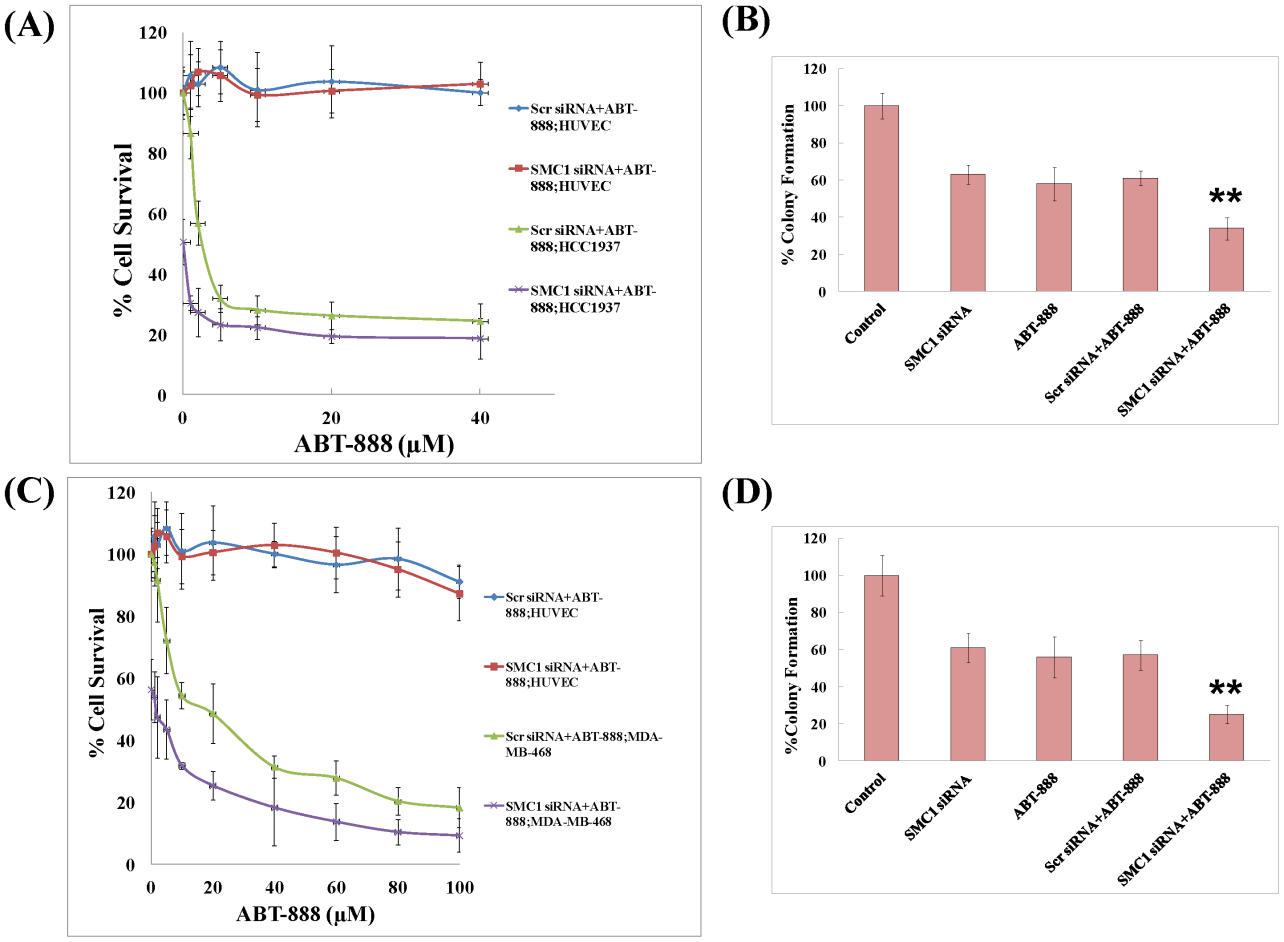
Effects of combining a PARP-inhibitor with SMC1 siRNA in TNBC basal-like cell lines. IC_50_ (half maximal inhibitory concentration) of the PARP-inhibitor, ABT-888 combined scrambled (scr) or SMC1 siRNA was tested in normal (HUVEC) and TNBC basal-like (MDA-MB-468, HCC1937) cell lines by MTT assay as detailed in methods section. Briefly, cells were transfected with scrambled siRNA or SMC1 siRNA (50 nM) using Lipofectamine RNAiMax following manufactures instructions and after 24 hours, transfected cells were treated with a range of ABT-888 (0–100 µM) and MTT assay was performed after 72 hours (**Panel A and C**). To further check the effect of SMC1 silencing on the efficacy of ABT-888, colony propagation assay was performed as described [37], [38]. Briefly, MDA-MB-468 and HCC1937 were transfected with scrambled or SMC1 siRNA (0.1×10^6^ cells/500 µl in triplicates). Aliquots of 50 and 100 µl (in triplicates) were taken in 60 mm size petri-dishes, separately, in a total volume of 4 ml with medium containing 20 µM ABT-888 (MDA-MB-468) and 5 µM (HCC1937). The medium was changed every 2 days and after 10 days, the cells were stained with 0.5% methylene blue and colonies were counted by Alpha Innotech Imager (**Panel B and D**). These results showed that SMC1 siRNA sensitized basal like TNBC cells irrespective of their *BRCA1* mutation status.

**Figure 6:**
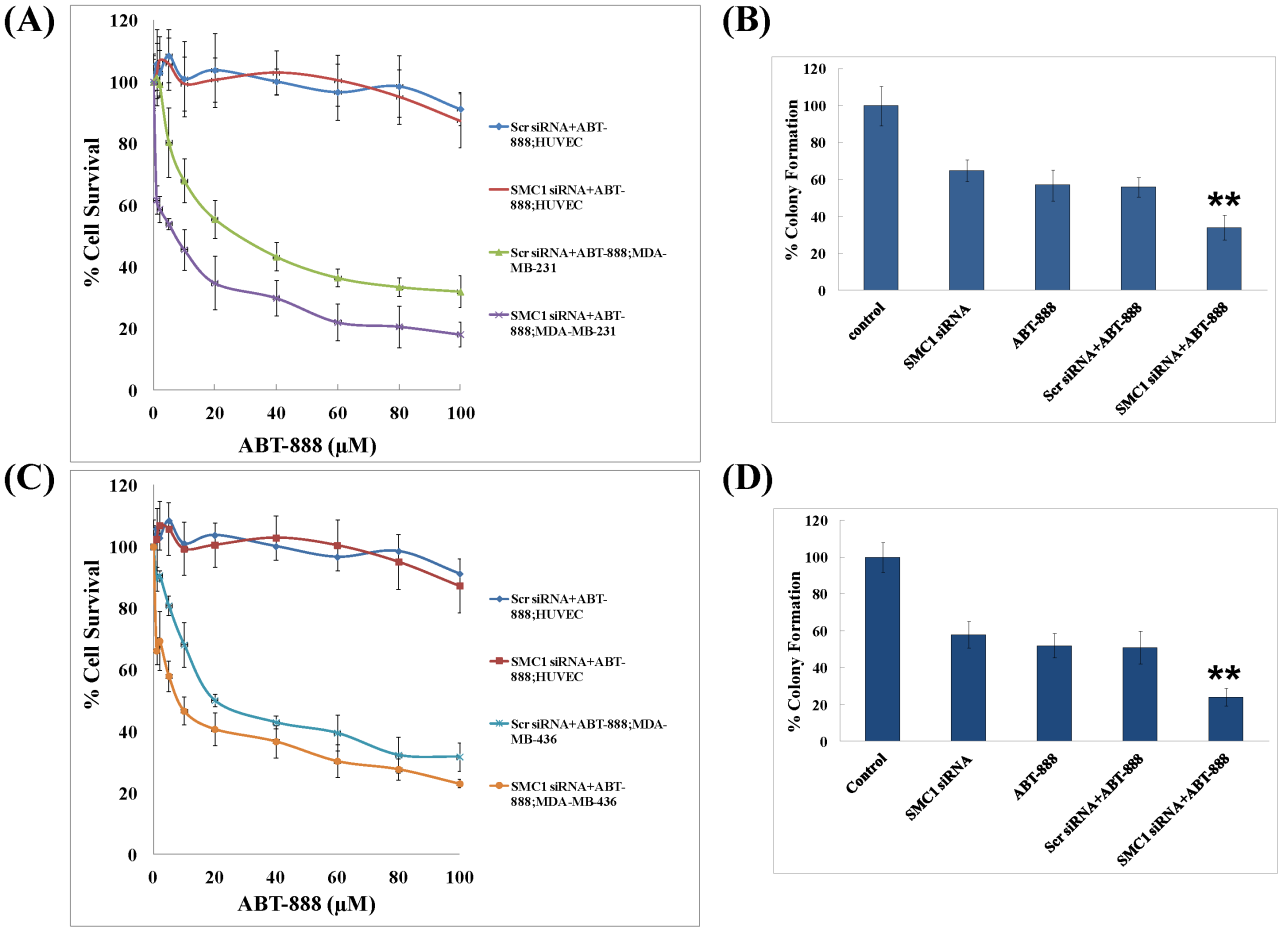
Effects of combining a PARP-inhibitor with SMC1 siRNA in TNBC mesenchymal stem-like cell lines. The effect of SMC1 siRNA was also tested on the mesenchymal stem-like TNBC cell subtype (MDA-MB-231, MDA-MB-436) which constitute the minor proportion of the total population of TNBC to explore the effect of SMC1 suppression on the sensitivity of the heterogeneous TNBC cells population towards PARP inhibitor. MDA-MB-231 (*BRCA1* wild type) and MDA-MB-436 (*BRCA1* mutated) cells were transfected with scrambled (scr) siRNA or SMC1 siRNA as described in the method section and after 24 hours, were treated with a range of ABT-888 (0–100 µM) and incubated at 37°C in CO_2_ incubator for 72 hours and survival of cells were determined by MTT assay. IC_50_ was calculated and the dose at which there was 50% cell death was used to determine the cell propagation capacity in presence of SMC1 siRNA by colony forming assay. Briefly, MDA-MB-231 and MDA-MB-436 cells were transfected with scrambled or SMC1 siRNA (0.1×10^6^ cells/500 µl in triplicates). Aliquots of 50 and 100 µl (in triplicates) were taken in 60 mm size petri-dishes, separately, in a total volume of 4 ml with medium containing 20 µM ABT-888. The medium was changed every 2 days and after 10 days, the cells were stained with 0.5% methylene blue and colonies were counted by Alpha Innotech Imager. These results showed that SMC1 siRNA sensitized both *BRCA1* functional and mutated mesenchymal stem-like TNBC cells toward ABT-888.

As triple negative breast cancer constitutes a highly heterogeneous cell population, the effect of SMC1 suppression by SMC1 siRNA (50 nM) combined with 0–100 µM ABT-888 was tested in both basal-like (MDA-MB-468, HCC 1937), and mesenchymal stem like (MDA-MB-231, MDA-MB-436) subtypes of TNBC cells. HUVEC cells were used as normal control. Our results showed that suppression of SMC1 by siRNA significantly sensitized both basal-like and mesenchymal stem-like subtypes, including in the *BRCA1* mutated TNBC lines towards ABT-888 (Fig. 5, Panel A and C; Fig. 6, Panel A and C). The IC_50_ of ABT-888 was 22.3±1.7 µM in MDA-MB-231, while with suppression of SMC1; IC_50_ of ABT-888 was 4.1±0.2 µM. Similarly, IC_50_ of ABT-888 was 19.2±1.9, 4.3±0.18 and 20.5±0.42 µM; with SMC1 suppression, IC_50_ was 2.1±0.17, 0.54±0.12 and 7.3±0.14 µM for MDA-MB-468, HCC1937 and MDA-MB-436 cells respectively. There was no significant effect of SMC1 siRNA seen in normal HUVEC line with addition of ABT-888. These results were further confirmed by the colony propagation assay (Fig. 5, Panel B and D; Fig. 6, Panel B and D). Our results shows that SMC1 suppression combined with ABT-888 sensitize the TNBC cells towards the PARP inhibitor including the *BRCA1* mutated cell lines.

## Discussion

The increased expression of SMC1 gene in triple negative breast cancer supports the idea that SMC1 biosynthesis is tightly regulated in normal cells and that an imbalance in the amount of this protein may directly affect the cell survival and other cellular functions [23]–[26]. Our studies demonstrate the presence of SMC1 in nucleus, which supports its role in chromosomal architecture and separation [15]–[21]; we also found SMC1 present in the cytosol and cell membrane along with SMC3. Although the role of SMC1 in the plasma membrane is not known, BRCA1, a SMC1 binding protein has recently shown to be involved in regulation of motility and migration of breast cancer cells [45]. Our results have shown for the first time that forced over-expression of SMC1 increases the wound healing capacity of TNBC cells, which indicate that SMC1 may be involved in the cell migration and tumor metastasis. A post-translationally modified form of SMC3 has shown to be present in the basement membrane and involved in cell adhesion [32], [33]. The role of SMC1 in basement membrane is currently not known, although it contains LRE (Leucine-Arginine-Glutamate) sequence for cell adhesion similar to SMC3 and other proteins directed to and involved in cell-cell and cell-matrix contacts [46], [47]. It is noteworthy that BRCA1 and SMC3 are present at the plasma membrane and basement membrane respectively and involved in the regulation of cell spreading and motility in cancer cells [32], [45]. Bamacan has also been identified in the exosomes purified from the pleural effusion of a breast cancer patient which may be related to their high concentration in malignant pleural fluid [48].

A number of targeted therapies have been studied in triple negative breast cancer, which causes a disproportionate number of breast cancer deaths due to heterogeneity of the tumors, their intrinsic aggressiveness and lack of treatment options. Recently, PARP inhibitors have been shown some activity in TNBC cells, but PARPs function as a DNA damage sensor for single and double stranded DNA breaks, and if PARP activity is blocked by homologous recombination, repair mechanisms kick in to protect the cells [12], [14]. BRCA1 and BRCA2 proteins are critical players in the homologous repair pathway; therefore the use of PARP inhibitors in BRCA-defective cancer cells is thought to lead to genetic damage and cell death by a synthetic lethal effect, which is borne out in clinical trials as PARP-inhibitors have shown more response in patients with *BRCA*-mutated breast cancers [12], [49]. There are now ongoing clinical trials looking at combinations of PARP-inhibitors alone and in combination with chemotherapy agents such as cisplatin and carboplatin in *BRCA*-mutated and wild type TNBC patients [50], [51].

**Figure 7:**
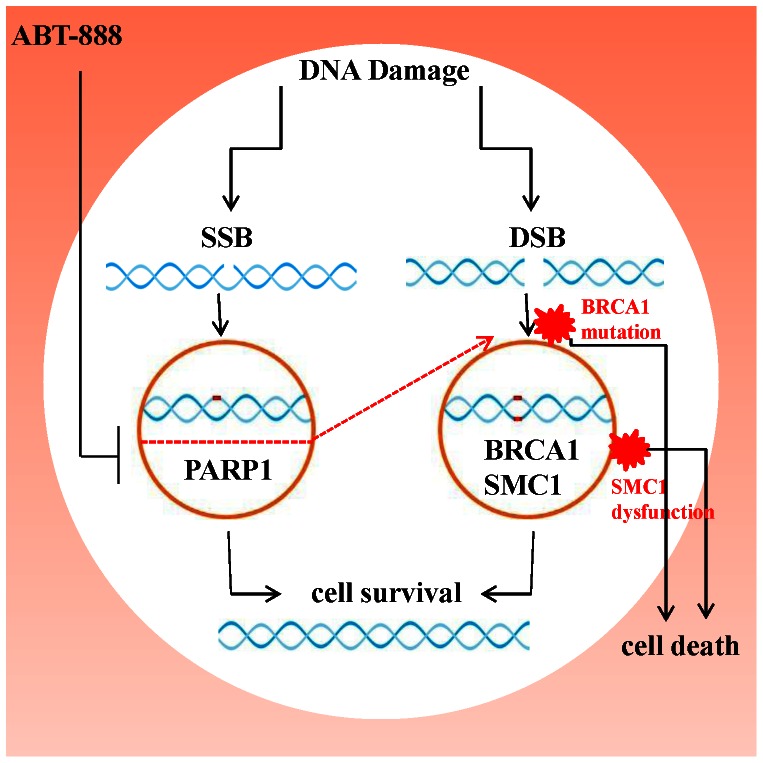
Schematic representation of role of SMC1 in sensitizing TNBC cells towards ABT-888. Targets of PARP inhibitor, ABT-888; PARP is depicted along with the *BRCA1* and SMC1. DSB, double-strand break; SSB, single-strand break; PARP, poly (ADP)-ribose polymerase; *BRCA1*, breast cancer 1, early onset; SMC1, structural maintenance of chromosome 1.

SMC1 has also been shown to bind with BRCA1 and together they are involved in the regulation of DNA damage response and cell cycle checkpoint-mediated repair [15]. It is possible that when present in excess, SMC1 acts in dominant-negative fashion with respect to the activity of the SMC1/SMC3/BRCA1 complex, affecting the efficacy of the complex towards various cellular functions. Therefore, overexpression of SMC1 in TNBC (including *BRCA* mutated tumors) may provide a target for pharmacological agents, as SMC1 inhibition may sensitize these cancer cells to PARP-inhibitors, cytotoxic chemotherapy, or both in combination (Fig. 7). Our results showed that when SMC1 expression was inhibited in combination with ABT-888 there was more than threefold increased sensitivity of ABT-888 towards both BRCA wild type and mutated TNBC cell lines including basal-like and mesenchymal stem-like subtypes. It has been previously shown that SMC1 siRNA treated colon cancer-derived cells exhibited reduced cell proliferation in response to another PARP-inhibitor, Olaparib [52]. This is in accordance with our findings that inhibiting SMC1 expression in TNBC cells via siRNA may increase their sensitivity to PARP-inhibitors. However, the mechanism of the PARP-cohesin interaction is not yet known, as PARP and SMC1 and other subunits of the cohesin complex have multiple functions that could be codependent. Further studies would be needed to determine if the inhibition of SMC1 is synergistic or additive with PARP-inhibitors in TNBC cell lines, including functional and mutated *BRCA*. Given SMC1 binding to *BRCA1* and its overexpression in TNBC, our results suggests that suppression of SMC1 may improve TNBC response rates to PARP-inhibitors either alone or in combination with chemotherapy. While PARP-inhibitors have shown the highest response rates in *BRCA*-mutated tumors in clinical trials, with addition of an SMC1 inhibitor, it may also be possible to improve their response rate in *BRCA* wild type triple negative breast cancer.
